# Effects of additive sensory noise on cognition

**DOI:** 10.3389/fnhum.2023.1092154

**Published:** 2023-06-01

**Authors:** Sage O. Sherman, Maya Greenstein, Mathias Basner, Torin K. Clark, Allison P. Anderson

**Affiliations:** ^1^Ann and H.J. Smead Department of Aerospace Engineering Sciences, University of Colorado, Boulder, Boulder, CO, United States; ^2^Unit for Experimental Psychiatry, Division of Sleep and Chronobiology, Department of Psychiatry, Perelman School of Medicine, University of Pennsylvania, Philadelphia, PA, United States

**Keywords:** stochastic resonance (SR), auditory white noise, noisy galvanic vestibular stimulation, cognition test battery for spaceflight, sensory cognition

## Abstract

**Background:**

Adding noise to a system to improve a weak signal’s throughput is known as stochastic resonance (SR). SR has been shown to improve sensory perception. Some limited research shows noise can also improve higher order processing, such as working memory, but it is unknown whether SR can broadly improve cognition.

**Objective:**

We investigated cognitive performance while applying auditory white noise (AWN) and/or noisy galvanic vestibular stimulation (nGVS).

**Methods:**

We measured cognitive performance (*n* = 13 subjects) while completing seven tasks in the cognition test battery (CTB). Cognition was assessed with and without the influence of AWN, nGVS, and both simultaneously. Performance in speed, accuracy, and efficiency was observed. A subjective questionnaire regarding preference for working in noisy environments was collected.

**Results:**

We did not find broad cognitive performance improvement under the influence of noise (*p* > 0.1). However, a significant interaction was found between subject and noise condition for accuracy (*p* = 0.023), indicating that some subjects exhibited cognitive changes with the addition of noise. Across all metrics, noisy environment preference may trend to be a potential indicator of whether subjects will exhibit SR cognitive benefits with a significant predictor in efficiency (*p* = 0.048).

**Conclusion:**

This study investigated using additive sensory noise to induce SR in overall cognition. Our results suggest that using noise to improve cognition is not applicable for a broad population; however, the effect of noise differs across individuals. Further, subjective questionnaires may be a means to identify which individuals are sensitive to SR cognitive benefits, but further investigation is needed.

## Highlights

- Additive sensory white noise does not broadly affect human cognitive performance.
- Influence of additive sensory noise on cognitive performance varies by individual.
- Cognitive effects of noise may be associated with a person’s noisy environment preference and warrants further investigation.

## Introduction

Stochastic resonance (SR) is a phenomenon where additive noise can improve the throughput of a signal in non-linear systems (Moss et al., 2004). Conceptually, SR may occur by applying an ideal level of noise, such that it resonates with the sensory signal. Therefore, it is believed that an “optimal” level of noise is required to achieve throughput enhancement (Galvan-Garza, 2018). Psychophysical experimentation suggests that SR can improve perceptual performance, such as lowering auditory thresholds, both within the same sensory modality [e.g., using auditory white noise (AWN) to improve hearing (Zeng et al., 2000)] and across separate sensory modalities [e.g., using noisy galvanic vestibular stimulation (nGVS) to improve visual perception (Voros et al., 2021)]. While perception has been shown to be affected by additive noise, there is limited research on whether higher order cognitive processes is subject to SR.

Noise-enhanced sensory information could be utilized by the whole central nervous system (Hidaka et al., 2000), suggesting that SR could affect higher order information processing. In human subject experiments, background AWN (∼78 dB SPL) improved verbal recall, visuo-spatial working memory, and motor response in inattentive school children (Söderlund et al., 2010; Helps et al., 2014). For a neurotypical population, AWN has been shown to improve elements of attention and visual/auditory working memory (Othman et al., 2019; Awada et al., 2022). Cognitive SR benefits extend to modalities other than auditory though. This is supported by studies showing that nGVS improves visual working memory in healthy adults (Wilkinson et al., 2008). Aside from enhanced processing in healthy adults, SR could also offset reduced perceptual abilities that may negatively affect cognition.

Bigelow and Agrawal (2014) summarized the link between vestibular and cognitive functions, noting that visuospatial ability and attention are negatively impacted in subjects with vestibular impairments. Pineault et al. (2020) also suggested that impairments to the saccule and semi-circular canals of the vestibular system affects various aspects of cognition. It has been shown that nGVS improves vestibular self-motion perception (Galvan-Garza, 2018; Keywan et al., 2019), suggesting enhanced vestibular function. Therefore, improved vestibular function from nGVS could potentially offset cognitive decrements due to perceptual impairment.

Existing studies that suggest adding sensory noise can enhance cognition are limited as they focus on specific cognitive domains and do not investigate cognitive effects more broadly. Cognitive domains are individual cognitive processes, like working memory, which are employed when synthesizing information for decision making and behavior control (Harvey, 2019). Certain cognitive domains recruit different regions of the brain (Basner et al., 2015) and additive noise influences regional activity within the cerebral cortex (Mendez-Balbuena et al., 2018; Huidobro, 2020). This regional influence may correspond to specific cognitive domains. For example, the temporal lobe houses auditory and other multisensory association areas in addition to cognitive centers that are attributed to memory (Kandel et al., 2000); thus, surrounding regions may see neuronal influence by AWN which could affect memory. Supporting this, Kim et al. (2013) reports that nGVS leads to gamma wave suppression in the frontal region which is associated with several cognitive abilities. However, literature investigating cognitive benefits of adding sensory noise has focused on working memory and motor response, neglecting other domains, such as vigilance and visual search. This presents a substantial gap in our knowledge of sensory noise influence on overall cognition as a thorough analysis across multiple cognitive domains has yet to be investigated.

Often, these studies also fail to consider the potential confounding effect of arousal induced by sensory stimulation as the mechanism of cognitive improvement, as opposed to the presumed mechanism of stochastic resonance. Arousal resulting from periodic visual and auditory stimuli have been shown to increase functional activity in frontal regions (Sturm and Willmes, 2001), potentially impacting cognitive abilities. Without the use of control conditions to assess the role of arousal from sensory stimulation, it is unclear whether SR is the dominant mechanism in any cognitive improvement.

Thus, our work aimed to explore the ability of enhancing broad cognitive performance using sensory noise. Our work evaluated cognition by using the validated cognition test battery (CTB) developed by Basner et al. (2015) which provides a sensitive evaluation of different cognitive domains using standardized techniques, such that when combined, the results provide a comprehensive insight on SR’s influence on cognition. We hypothesized that single modality noise (AWN and nGVS) would enhance cognitive performance in human subjects when compared to performance without noise. Further, we hypothesized that stimulating both modalities simultaneously to induce multi-modal SR (MMSR) would enhance performance to a greater degree than single modality alone. This hypothesis is novel as, to our knowledge, no investigation exists evaluating the mental performance effects of compounding sensory noise across multiple modalities. To address the gap associated with improvement due to arousal, our work investigates the role of additive noise versus simple arousal stimulation in influencing cognition.

We also investigated the degree to which we could identify whether subjects may be sensitive to SR cognitive performance enhancement. SR perception studies have suggested that some individuals are susceptible to SR perception improvements, while others are not (Ries, 2007; Galvan-Garza, 2018). Thus, we hypothesized that only some subjects may receive SR cognitive benefits. Currently, there is no way to predict *a priori* whether an individual is likely to be sensitive to SR performance improvement. Therefore, we developed a subjective questionnaire for subjects to rate how well they could maintain focus in quiet and noisy environments. We hypothesized that there would be a positive correlation between noisy environment preference and cognitive enhancement under the influence of added sensory noise.

## Materials and methods

### Subjects

Thirteen subjects (7F/6M, range = 20–40 years, mean = 29.5 years, SD = 6.6 years) completed testing in the Bioastronautics Lab at the University of Colorado-Boulder. An *a priori* power analysis based on the results of Wilkinson et al. (2008) and Söderlund et al. (2010) suggested that we needed 8–12 subjects for our study design to find an effect size greater than 0.3, which was expected based on the former’s findings. This research was approved by the University of Colorado-Boulder’s Institutional Review Board (#20-0419) and written informed consent was obtained prior to participation. Subjects were pre-screened and excluded if they reported a history of health issues that could impact cognitive abilities, such as severe head trauma or disorders associated with thinking impairment. They were also excluded if they reported health issues that could impact auditory or vestibular processing, such as language impairment or vestibular dysfunction. Additionally, subjects underwent auditory screening to verify healthy and unobstructed ear canals (via otoscopy), normal tympanometry, and normal hearing (audiometric thresholds < = 25 dB HL up to 8 kHz).

### Protocol and study design

Broadband AWN was administered to subjects through ear buds (Essential Earphones HD) and a Samsung Tablet A; the auditory profiles were developed and calibrated by Creare LLC (Hanover, NH, United States). Broadband, unipolar, zero-mean white noise was bilaterally administered to subject mastoids through the Galvanic Vestibular Oscillating Stimulator (model 0810, Soterix Medical, Woodbridge, NJ, United States) using electrodes with a contact area of 2 cm^2^. Tasks were completed using a Dell Latitude E6430 laptop, which is specifically calibrated to run the CTB, in a single walled sound booth (Whisperoom, Knoxville, TN, United States, MDL 4872).

A within-subject experimental design was implemented. Seven tasks in the CTB were chosen as they are associated with distinct cognitive domains and recruit different regions in the brain, allowing us to explore cognition and its sub-domains in a manner far more comprehensively than has been found in the literature. These seven tasks are presented in Table 1 [recreated from Basner et al. (2015)], summarizing each task’s cognitive domain and areas of the brain recruited to complete the task.

**TABLE 1:** Cognitive domains and brain regions associated with the seven CTB tasks (Basner et al., 2015).

Task	Cognitive domains	Recruited brain regions
Digit symbol substitution (DSST)	Visual search/Spatial memory/Paired associate learning	Temporal cortex/Prefrontal cortex/Motor cortex
Line orientation (LOT)	Spatial orientation	Right temporo-parietal cortex/Visual cortex
Matrix reasoning (MRT)	Abstract reasoning	Prefrontal cortex/Parietal cortex/Temporal cortex
Fractal 2-back (F2B)	Working memory	Dorsolateral prefrontal cortex/Cingulate/Hippocampus
Motor praxis (MPT)	Sensory-motor speed	Sensorimotor cortex
Psychomotor vigilance (PVT)	Vigilant attention	Prefrontal cortex/Motor cortex/Inferior parietal and visual cortex
Visual object learning (VOLT)	Spatial learning/Memory	Medial temporal cortex/Hippocampus

In their initial visit, subjects were trained in the standard manner on the CTB tasks by watching a 20-min tutorial video, after which they completed two practice trials of each CTB task. Next, testing occurred across two subsequent visits, where subjects completed all testing for each specific CTB task within a single session. MRT, MPT, and PVT were tested in one session and DSST, LOT, F2B, and VOLT were tested in the other session. The order of the tasks within the test day was randomized for each of the two test days.

In the CTB, cognitive performance is quantified in terms of speed and accuracy. However, the speed-accuracy tradeoff is confounding when evaluating improved performance (Wicklegren, 1977). The literature accounts for this through a post-hoc combination of the normalized speed and accuracy metrics, which is often referred to as efficiency (Scully et al., 2019; Basner et al., 2021). The dependent variables of accuracy, speed, and efficiency were used to assess performance in the cognitive tasks.

Recall, it is thought there is an optimal level of noise in terms of producing SR-benefits that depends on the subject, task, and sensory system (Moss et al., 2004). Thus, for each CTB task, a range of AWN and nGVS levels were assessed for each subject. Four nGVS levels [(0.2, 0.4, 0.6, and 0.8 mA)] and three AWN levels [(40, 55, and 70 dB SPL)] were tested in a randomized order, as has been done in our prior work (Voros et al., 2021). Speed and accuracy were corrected to account for trial-specific differences and learning effects, using corrections from Basner et al. (2020). From this initial set of measures, the SR level yielding the best score in feedback, another measure of combined performance in the CTB, was selected as the subject-specific best (or close to subject optimal) AWN and nGVS levels.

Once the subject-specific best SR levels were identified, six experimental conditions were investigated to understand the effects of additive noise on cognition. Subject-specific best levels of AWN, nGVS, and MMSR were tested, as determined from the initial suite of measures. To investigate the potentially confounding effect of arousal, subjects completed tasks under the stimulation of suprathreshold stimuli−an auditory pure tone signal at 55 dB SPL, as well as a direct current GVS (DC GVS) signal at 0.8 mA. These conditions stimulate the sensory modalities with a non-random signal in a manner that would not induce SR benefits, but would cause arousal. We hypothesized that these stimulation control conditions would not result in significant performance changes from sham, if indeed the mechanism for any benefit from added noise is due to SR. To summarize, the following six conditions were retained for statistical analysis: three control conditions; no stimulation sham, 55 dB pure tone auditory stimulation, and 0.8 mA DC GVS stimulation and three noise conditions; subject-specific best AWN, best nGVS, and MMSR. All conditions were presented and tested in a randomized order for the cognitive tasks within each of the two test sessions. Short breaks were provided between tests to help mitigate subject mental fatigue, but as in other studies using nGVS (Goel et al., 2015; Mulavara et al., 2015; Galvan-Garza, 2018; Inukai et al., 2018; Keywan et al., 2018, 2019; Voros et al., 2021) we did not employ a more extensive break between nGVS applications, since the most rigorous studies using nGVS have not found carryover effects between nGVS stimulation levels (Nooristani et al., 2019; Keywan et al., 2020).

After completing all cognitive testing, subjects completed a subjective five-point Likert scale questionnaire that asked how well they felt they could maintain focus in quiet and noisy environments. Their noisy environment preference score was defined as the difference in subject ranking between quiet and noisy environments (i.e., a negative score means the subject prefers working in quiet places and a positive score means they prefer working in noisy places). This survey can be found in Supplementary Data Sheet 1.

### Analysis

A within-subjects analysis was completed for the metrics of accuracy, speed, and efficiency. Two separate analyses were done by comparing sham to the noise conditions and to the stimulation control conditions. In each analysis, performance outcomes on each of the 7 CTB tasks were collapsed into one scale to create a comprehensive cognition metric. For this comprehensive metric, data was initially adjusted by subtracting the subject’s specific average across the conditions of interest in that CTB task, to account for individual differences in performance. From there, the data was standardized for the task by calculating the z-score of each measurement with respect to all measurements across subjects within that CTB task as shown in Equation 1. *Z_i_* represents the standardized cognition metric and *P_i_* is the raw scores of that task datapoint. *M_T_* and σ_*T*_ were the mean and standard deviation, respectively, of all raw data in the specific task. This process has been done for CTB data in prior work (Scully et al., 2019), yielding a normalized cognition outcome.


(1) $$Z_{i}=\frac{P_{i}-M_{T}}{\sigma_{T}}$$

A repeated measures ANOVA (RMANOVA) with four levels (sham, AWN, nGVS, and MMSR) was conducted to investigate the effect of noise on cognition using this normalized cognition metric. This was applied to each of the metrics of speed and accuracy. Efficiency was calculated as the mean of these two normalized metrics. A separate RMANOVA was also completed for the three control conditions (sham, tone, DC GVS) to investigate the effect of arousal on cognition. Assumptions for homogeneity and residual normality were tested to ensure that parametric statistics were appropriate. Datapoints that created semi-studentized residuals greater than three were removed as outliers. If there was an outlier in one metric, say speed, the associated datapoint was also removed from the other two metrics, accuracy and efficiency. If the F-test results from the RMANOVAs were significant, Tukey HSD multiple pairwise comparisons were used to identify which conditions were different from another. If the F-test results from the RMANOVAs were insignificant, an equivalence test was completed to indicate whether the conditions were equivalent following the methods conducted by Rusticus and Lovato (2011).

To assess noise effects on overall cognition, as per our first hypothesis, subjects were treated as a random effect in our RMANOVAs, allowing us to posit on the broad utility of additive noise across all subjects in our sample. This analysis was done for the noise conditions (sham, nGVS, AWN, and MMSR) and control conditions (sham, pure tone auditory stimulation, DC GVS). For the second hypothesis analysis, subjects were included as an interaction term along with the noise conditions, allowing us to posit on whether noise effects are different across individuals.

Additionally, an exploratory analysis was conducted to see whether subjective noisy environment preference could be an indicator for individual differences in noise effects on cognition. Subjects’ normalized cognition metric in the sham condition was subtracted from their normalized cognition metric in the additive noise conditions. The calculation of this metric is found in Equation 2. Linear models were fit to this entire dataset against their noisy environment preference scores.


(2) $$\Delta \,Z_{i}=Z_{i,n\,o\,i\,s\,e}-Z_{i,s\,h\,a\,m}$$

## Results

**FIGURE 1:**
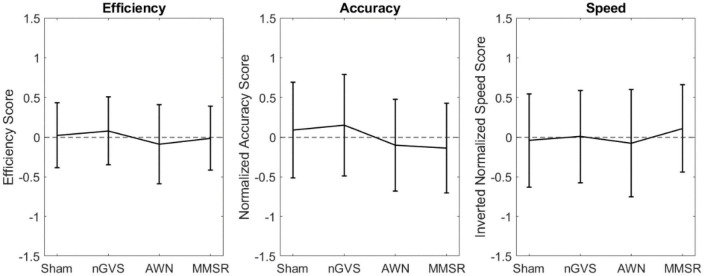
Scatter plots compiling normalized cognition metric scores combined across CTB tasks, for the noise conditions and sham. Error bars indicate the 95% confidence interval for score in that condition.

For all models presented in these results, there were no observable violations of the residuals from our assumptions. Figure 1 compares the normalized cognition metric scores for the noise conditions and the sham condition, for all subjects and tasks. This figure represents the difference in overall cognition, where higher scores in efficiency, accuracy, and inverted speed imply better performance. Table 2 displays the RMANOVA results with subjects included as a random effect. Contradictory to our hypothesis that additive noise would improve cognition, no significant differences were found between sham and the noise conditions for all metrics. Separate to this main comprehensive analysis, we conducted an exploratory analysis of accuracy and speed in each CTB task individually and found no significant differences. These results are found in Supplementary Table 1 and Supplementary Figure 1 with figures and statistical findings. For all tables presented in these results, F(#, #) denotes the degrees of freedom (DOF) of our Omnibus tests, they are represented as F(DOF for treatment factor, DOF for error).

**TABLE 2:** 1×4 RMANOVA results for sham and noise conditions.

Metric	F(3, 343)	*P*-value	$\eta_{p}^{2}$
Efficiency	1.08	0.357	0.009
Accuracy	2.09	0.101	0.018
Speed	0.54	0.654	0.005

Subjects included as a random effect.

Five outliers were identified and removed within this first model, out of 364 total data points. When all of the data was included in the RMANOVA, the *p*-values increased. Thus, the conclusion that noise does not significantly affect cognition metric scores remains the same. No outliers were identified or removed in the other models presented.

**FIGURE 2:**
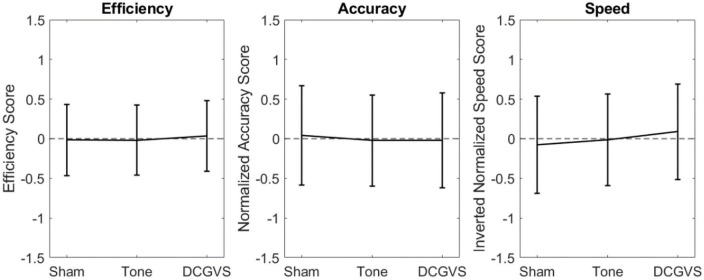
Scatter plots compiling normalized cognition metric scores combined across CTB tasks for the control conditions. Error bars indicate the 95% confidence interval for score in that condition.

To assess the effect of arousal, the same RMANOVA analysis was applied to the control conditions. These results are found in Figure 2 and Table 3. In agreement with our hypothesis that arousal stimulation alone would not impact cognition, no significant differences between the control conditions were identified.

**TABLE 3:** 1×3 RMANOVA results for the control conditions.

Metric	F(2, 258)	*P*-value	$\eta_{p}^{2}$
Efficiency	0.14	0.866	0.001
Accuracy	0.11	0.893	0.001
Speed	0.6	0.547	0.005

Subjects included as a random effect.

The lack of significant differences was further evaluated using a series of equivalence tests. First, leveraging the data from Wilkinson et al. (2008), a 90% equivalence interval of ± 0.793 was defined for the difference between noise conditions. When comparing the noise conditions to sham, the largest 95% confidence interval for the multiple comparison was the mean difference ± 0.263 for efficiency, while for accuracy and speed it was ± 0.369. The small confidence intervals in our data suggest that the efficiency, accuracy, and speed were all equivalent between the noise treatments and sham. The largest 95% confidence interval for the control condition multiple comparison in our data (Figure 2) was only ± 0.348, suggesting the performance between the control conditions were equivalent.

**FIGURE 3:**
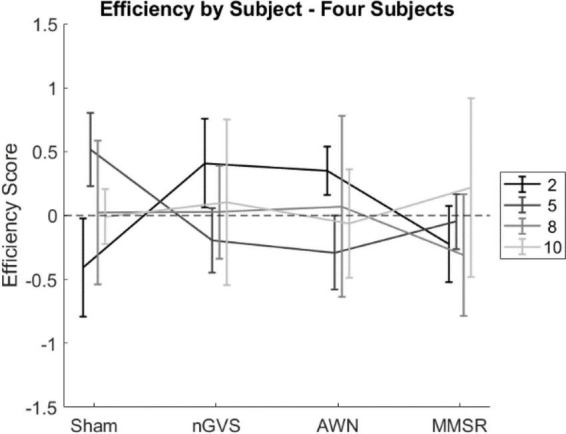
Scatter plots compiling efficiency scores across CTB tasks for four individual subjects for the noise conditions. Error bars indicate the 95% confidence interval for score in that condition.

To evaluate whether noise effects depend on subject (second hypothesis), we investigated the interaction of subject and condition. These results are presented in Table 4. In agreement with our hypothesis, significant interactions between subject and noise condition were identified for accuracy, but not speed and efficiency. This suggests that noise effects on cognition are inter-individually dependent. The efficiency results of four subjects are illustrated in Figure 3, where subject 2 appears to have cognitive benefits from applying noise, subject 5 was hindered, and subjects 8 and 10 have varied performance independent of noise. Four subjects are shown for legibility, plots containing all subjects can be found in Supplementary Figure 2.

**TABLE 4:** 1×4 RMANOVA results for the sham and noise conditions.

Metric	F(36, 312)	*P*-value	$\eta_{p}^{2}$
Efficiency	1.66	0.097	0.134
Accuracy*	1.58	0.023	0.154
Speed	1.07	0.364	0.110

Subjects included as an interaction term. Asterisks represent metrics that met a statistical significance below 0.05.

**FIGURE 4:**
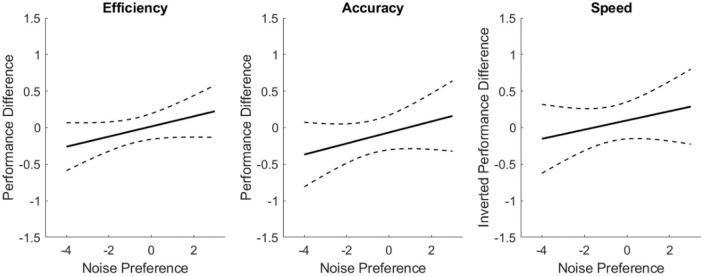
Linear regressions of cognitive performance improvement from sham as a function of noisy environment preference. Dashed lines indicate the 95% confidence interval of the modeled fit.

Figure 4 explores the mental performance difference (normalized sham cognition metric subtracted from normalized noise cognition metric) as a function of noisy environment preference. These linear models use data from all noise conditions, independent of sensory modality, to assess trends of user susceptibility to noise given preference. The characteristics of these models are presented in Table 5. Positive slopes were identified for all three metrics (inverting speed so positive implies performance improvement). These trends, while consistent with the hypothesis, were not statistically significant for the metrics of accuracy and speed, but was statistically significant for efficiency. The slope of the regression line indicates a change in effect across the scale with an effect size of 0.44 for speed, 0.53 for accuracy, and 0.48 for efficiency.

**TABLE 5:** Statistical results for linear regressions of cognitive performance improvements as a function of noisy environment preference.

Metric	Slope	*P*-value
Efficiency	0.069	0.048
Accuracy	0.075	0.109
Speed	0.063	0.212

Models fit to all noise condition data.

## Discussion

This research aimed to understand the utility of using additive sensory noise to improve overall cognition. To our knowledge, this research represents the most comprehensive assessment of the effects of SR noise on cognition. We assessed performance across a broad range of cognitive domains. We also incorporated an expansive set of control conditions to investigate arousal effects. This investigation was similar to cross-modality perception studies which found noise, not arousal, was the mechanism of perceptual enhancements (Lugo et al., 2008). Further, we investigated mental performance effects of compounding sensory noise across multiple modalities.

This work observed subject performance in seven tasks of the CTB while under the influence of nGVS, AWN, and MMSR. Observing performance metrics of efficiency, accuracy, and speed for the cognitive tasks in our broad population, no significant level differences were found between any of the conditions. Additive sensory stimulation, whether noisy (aimed at inducing SR), multi-modal, or control stimulations (pure tone auditory or DC GVS), had no significant effect on broad cognitive performance, neither beneficial nor degrading. Visually though, there appears to be larger performance differences between the noise conditions and sham (Figure 1) than there were for the control conditions (Figure 2). This may suggest that random noisy sensory stimulation influences cognition to a greater degree than non-noisy stimulation.

While previous working memory studies using nGVS were able to find significant differences with small subject numbers (Wilkinson et al., 2008), these studies, were limited in that they explored a singular aspect of cognition. While in this study we investigated 13 individuals, our methods comprehensively investigate seven tasks related to cognitive processing, which could allow us to understand broad effects of noise on cognition. Additionally, the repeated observations of our subjects in different tasks increased the statistical power of our models. Our results indicate via a retrospective power analysis that 12 subjects are sufficient to identify significant interaction differences, as shown in Table 4. However, based on the η^2^ of the noise condition term alone (Table 2) it was found that 100 subjects are needed to reach significant effect. Thus, this suggests that individual differences may be the dominant effect of SR, rather than broad cognitive benefits across all individuals. Individualized responses to sensory noise to improve cognition may be consistent with the findings of Helps et al. (2014) which found that only children with low attention tendencies cognitively benefitted from loud auditory white noise (≥ 65 dB). On the other hand, our results contrast those of Othman et al. (2019) and Awada et al. (2022) using AWN and Wilkinson et al. (2008) using nGVS to improve working memory, both in healthy adults. It could be possible that the benefits of additive sensory noise are limited to working memory (or other specific cognitive domains) and do not yield broad cognitive benefits, like we assessed here using seven tasks from the CTB. However, we conducted an exploratory RMANOVA of the adjusted scores for the fractal 2-back, a working memory task, and it still showed insignificance as well (*p* > 0.3). As such, our specific working memory task evaluation contradict findings in the literature. Referencing our exploratory analysis in Supplementary Table 1 and Supplementary Figure 1, we found no significant differences in each of the individual tasks. It should be noted that our findings supplement mixed results within the literature when investigating the role of auditory noise in cognition for a neurotypical group. Awada et al. (2022) found evidence that certain noise levels improved aspects of attention and working memory; however, not all cognitive tests evaluated or noise levels administered yielded significant improvements from ambient noise. This could suggest that noise does not influence neurotypical individuals to the degree it influences those with attentional disorders. While our results are not promising for generally using noise to enhance cognition, our results do indicate that individuals may be susceptible to benefits. Future work may move beyond inferential statistics used herein to analyze differences with Bayesian methods, which could provide further indications on the effect of noise on cognitive processes. Additionally, while we used the guidance of Basner et al. (2020) to correct for longitudinal effects that can stem from task learning or fatigue, it was of concern that these effects could still impact our results. Thus, we completed a regression analysis of accuracy and speed performance against number of times the task was completed to confirm this was not the case. We found no significant trends to indicate longitudinal effects skewed our data. These results can be found in Supplementary Figures 3, 4. Along with this sanity check, we believe our randomization procedures made us robust to this experimental concern.

The results of the linear models on preference for working in noisy environments shows novel promise for identifying users that may effectively use sensory noise for improved cognitive performance in an SR manner. Experimental literature suggests that some individuals are perceptual SR exhibitors, while others are not influenced by additive noise (Ries, 2007; Galvan-Garza, 2018). There has not been a way to identify, *a priori*, whether someone is susceptible though. This work identified trends in correlating cognitive performance improvement with subjective preference for working in noisy environments. A statistically significant, positive relationship was found between the efficiency metric and noise preference. The slopes of speed and accuracy trended toward significance, showing that additive sensory noise increases accuracy and reduces speed for subjects that prefer working in noisy environments. While there remains variability in response across the seven CTB tasks, these findings indicate that for some subjects, additive noise may yield improvements in cognitive performance, via the mechanism of SR. Further, those individuals were able to self-identify as performing better in noisy settings. To our knowledge, this is the first investigation exploring a means to independently identify which individuals may be susceptible to exhibiting SR benefits. This work’s brief noise preference questionnaire points toward working environment affinity as a potential indicator for finding individuals that could see cognitive enhancement from additive noise. The role of individual differences in preference toward working in noisy environments and SR exhibition, particularly in cognitive performance, warrants further investigation.

We want to note two limitations to this study. First, while we utilized a power analysis to guide the number of subjects we tested, thirteen is still a small sample size. This could explain why we were able to find a significant trend for noisy environment preference in efficiency, while we were not able to find significant main effects in our other analyses. However, based upon the effect sizes we observed, the population-wide effects of applying auditory or vestibular white noise on cognition appear quite small and may not be practically relevant. Second, we also note that the repeated application of nGVS (or AWN, DC GVS, or pure tone auditory stimulation) potentially could have long-term effects on cognition. While the literature does not indicate these carryover effects are anticipated, it also has not been rigorously evaluated, as has been done for other neuromodulation techniques (Medeiros et al., 2012; Keywan et al., 2020). This should be investigated in the future to assess the degree to which carryover effect may be found in our study.

## Conclusion

This investigation applied a comprehensive and rigorous evaluation of using sensory noise to improve cognition using a suite of standard cognitive tests and performance comparisons with stimulation control conditions. We conclude that applying additive noise to the auditory and vestibular sensory modalities, as well as to both simultaneously, will not result in improved cognitive performance for a broad population. However, our results indicate that additive noise may have differing cognitive effects across individuals. We assessed a subjective survey’s applicability to identify these individuals based on reporting of preference for working in a noisy environment and found a statistically significant, positive relationship. Thus, this type of subjective reporting may be a useful indicator, but further research into other identifying questions or techniques is needed.

## Data availability statement

The raw data supporting the conclusions of this article will be made available by the authors, without undue reservation.

## Ethics statement

The studies involving human participants were reviewed and approved by the University of Colorado, Boulder’s Institutional Review Board. The patients/participants provided their written informed consent to participate in this study.

## Author contributions

SS is the primary author and researcher on this manuscript. MG helped in the development and execution of this experiment. MB allowed us to use the cognition test battery and provided critical feedback. TC and AA were principal investigators on this project and provided critical feedback. All authors contributed to the article and approved the submitted version.
