# Supplementary material for: Effects of additive sensory noise on cognition
Source: Front Hum Neurosci. 2023 Jun 1;17:1092154. doi: 10.3389/fnhum.2023.1092154 (PMC10270290; doi:10.3389/fnhum.2023.1092154)
Supplement: Supplementary file 1 [file Table_1.docx]

Appendix B1: Table of RMANOVA Results for Individual Tasks

|  | **Accuracy** | | **Speed** | |
| --- | --- | --- | --- | --- |
| **Test** | **F(3,36)** | **P-Value** | **F(3,36)** | **P-Value** |
| Digit Symbol Substitution (DSST) | 0.23 | 0.874 | 1.75 | 0.174 |
| Line Orientation (LOT) | 1.42 | 0.253 | 0.3 | 0.827 |
| Motor Praxis (MPT) | 0.2 | 0.893 | 1.9 | 0.147 |
| Matrix Reasoning (MRT) | 0.13 | 0.940 | 0.66 | 0.582 |
| Fractal 2-Back (F2B) | 1.15 | 0.344 | 0.51 | 0.677 |
| Psychomotor Vigilance (PVT) | 0.52 | 0.673 | 0.63 | 0.602 |
| Visual Object Learning (VOLT) | 0.57 | 0.639 | 1.47 | 0.24 |
