# Supplementary material for: Effects of additive sensory noise on cognition
Source: Front Hum Neurosci. 2023 Jun 1;17:1092154. doi: 10.3389/fnhum.2023.1092154 (PMC10270290; doi:10.3389/fnhum.2023.1092154)
Supplement: Supplementary file 2 [file Table_2.docx]

Appendix D: Table of Model Fit Results for Individual Tasks in Accuracy and Speed

|  | **Accuracy** | | **Speed** | |
| --- | --- | --- | --- | --- |
| **Test** | **Slope** | **P-Value** | **Slope** | **P-Value** |
| Digit Symbol Substitution (DSST) | -0.02 | 0.673 | 1.75 | 0.638 |
| Line Orientation (LOT) | -0.09 | 0.880 | -32.9 | 0.402 |
| Motor Praxis (MPT) | 0.46 | 0.090 | 3.02 | 0.372 |
| Matrix Reasoning (MRT) | -0.24 | 0.372 | 68.4 | 0.095 |
| Fractal 2-Back (F2B) | -0.25 | 0.071 | -1.2 | 0.540 |
| Psychomotor Vigilance (PVT) | -0.07 | 0.557 | -0.41 | 0.430 |
| Visual Object Learning (VOLT) | -0.40 | 0.054 | 1.6 | 0.836 |
