# Supplementary material for: Effects of additive sensory noise on cognition
Source: Front Hum Neurosci. 2023 Jun 1;17:1092154. doi: 10.3389/fnhum.2023.1092154 (PMC10270290; doi:10.3389/fnhum.2023.1092154)
Supplement: Supplementary file 3 [file Data_Sheet_1.docx]

Appendix A: Hearing Questionnaire

To what extent, can you maintain focus while working in settings with background noise (ie. coffee shops)?

1. To a very small extent

2. To a small extent

3. To a moderate extent

4. To a large extent

5. To a very large extent

To what extent, can you maintain focus while working in silent environments?

1. To a very small extent

2. To a small extent

3. To a moderate extent

4. To a large extent

5. To a very large extent
