# Supplementary figures and images for: Effects of additive sensory noise on cognition
Source: Front Hum Neurosci. 2023 Jun 1;17:1092154. doi: 10.3389/fnhum.2023.1092154 (PMC10270290; doi:10.3389/fnhum.2023.1092154)

## Appendix B2: Figure Illustrations for Performance in Individual Tasks

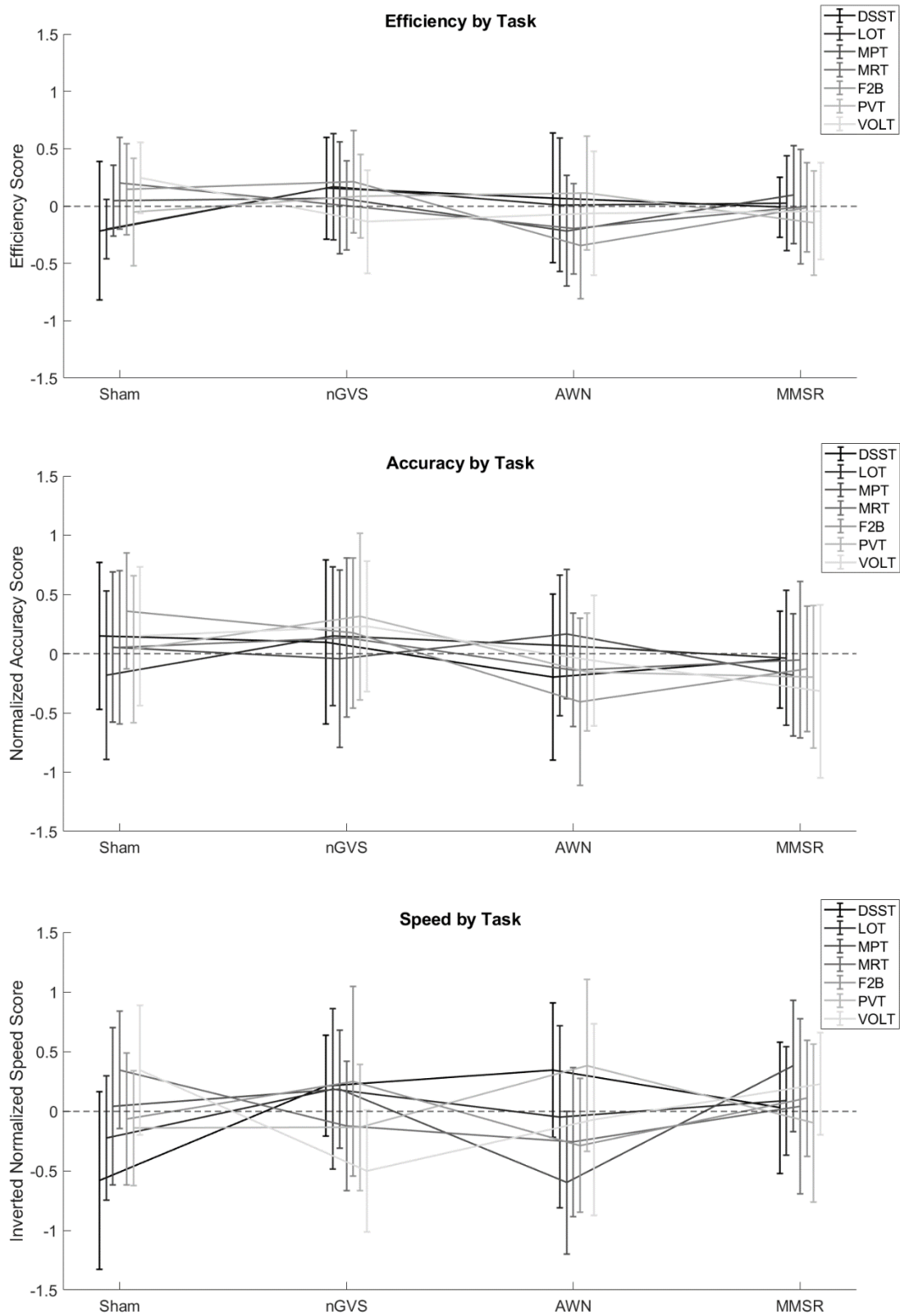

Supplement: Supplementary file 4 [file Image_1.pdf]

## Appendix C: Figure Illustrations for Performance in Individual Subjects

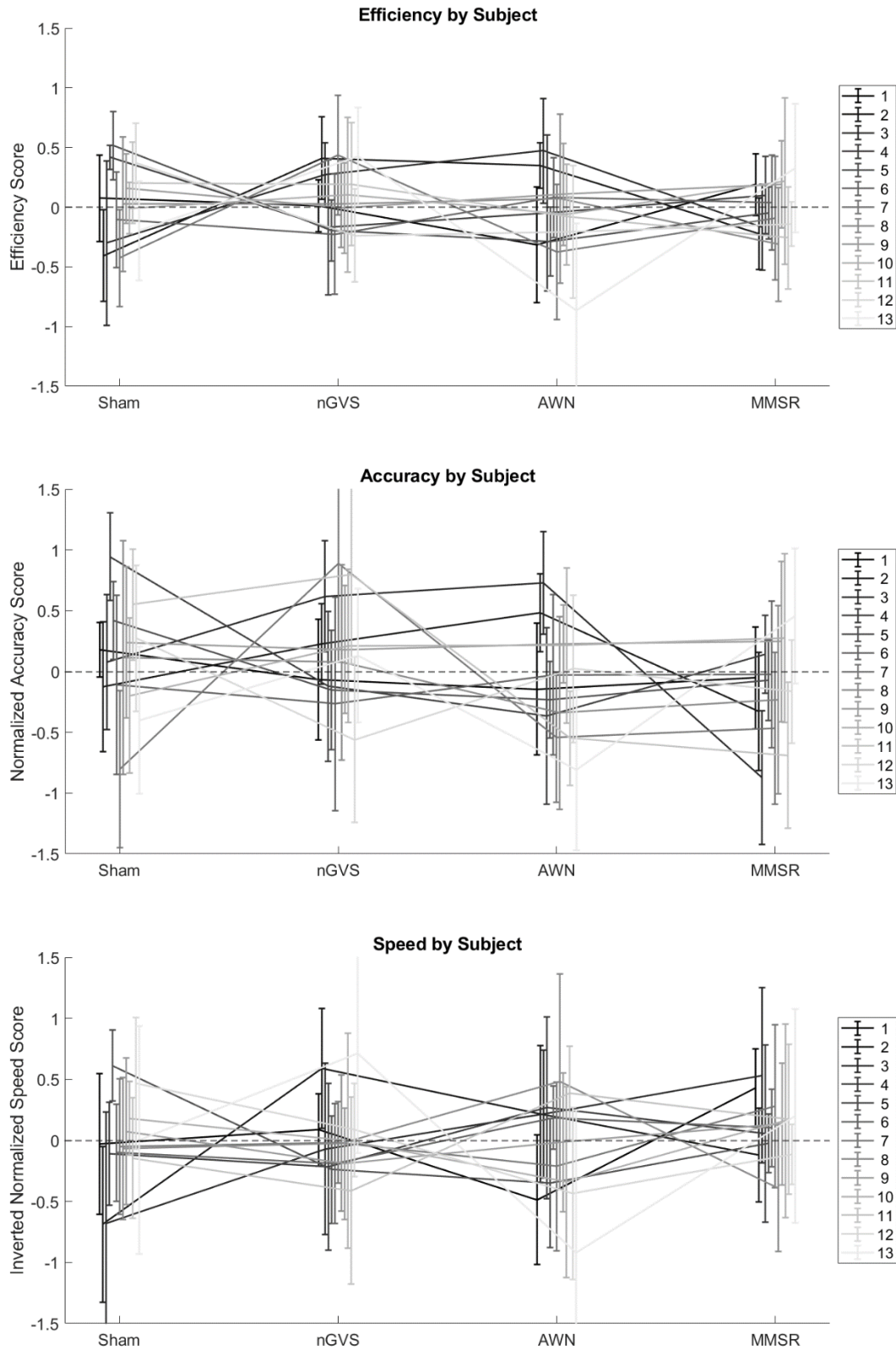

Supplement: Supplementary file 5 [file Image_2.pdf]
