# Supplementary material for: Effects of additive sensory noise on cognition
Source: Front Hum Neurosci. 2023 Jun 1;17:1092154. doi: 10.3389/fnhum.2023.1092154 (PMC10270290; doi:10.3389/fnhum.2023.1092154)

Appendix D3: Illustrations for Speed Performance by Task Administration. Error bars represent the standard error of the mean.

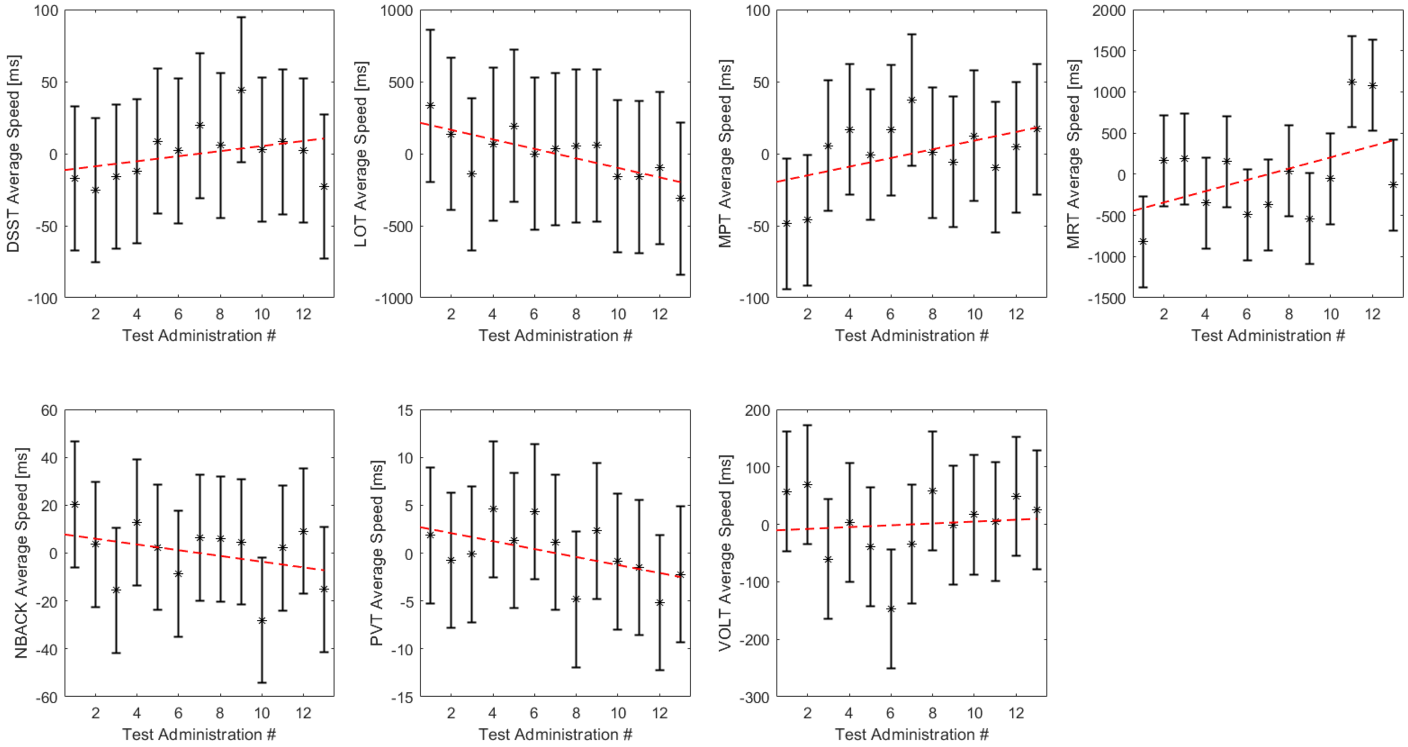

Supplement: Supplementary file 7 [file Image_4.pdf]
